# Supplementary material for: Alignment of spatial transcriptomics data using diffeomorphic metric mapping
Source: bioRxiv. 2023 Apr 12:2023.04.11.534630. Preprint. [Version 1] doi: 10.1101/2023.04.11.534630 (PMC10120659; doi:10.1101/2023.04.11.534630)
Supplement: Supplementary file 1 [file NIHPP2023.04.11.534630v1-supplement-3.pdf]

bioRxiv preprint doi: https://doi.org/10.1101/2023.04.11.534630; this version posted April 12, 2023. The copyright holder for this preprint (which was not certified by peer review) is the author/funder, who has granted bioRxiv a license to display the preprint in perpetuity. It is made available under aCC-BY 4.0 International license.

Supplementary Figures

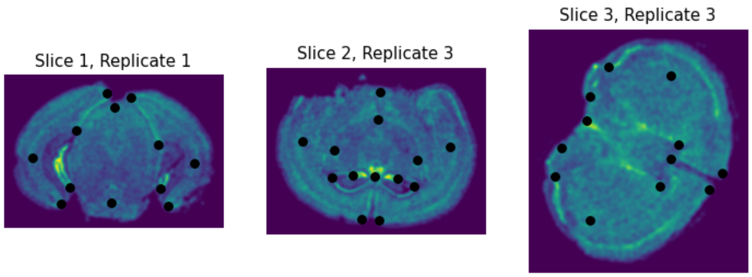

Supplemental Figure 1. Manually placed landmark locations on ST datasets for one representative biological replicate spanning 3 different locations with respect to bregma.

bioRxiv preprint doi: https://doi.org/10.1101/2023.04.11.534630; this version posted April 12, 2023. The copyright holder for this preprint (which was not certified by peer review) is the author/funder, who has granted bioRxiv a license to display the preprint in perpetuity. It is made available under aCC-BY 4.0 International license.

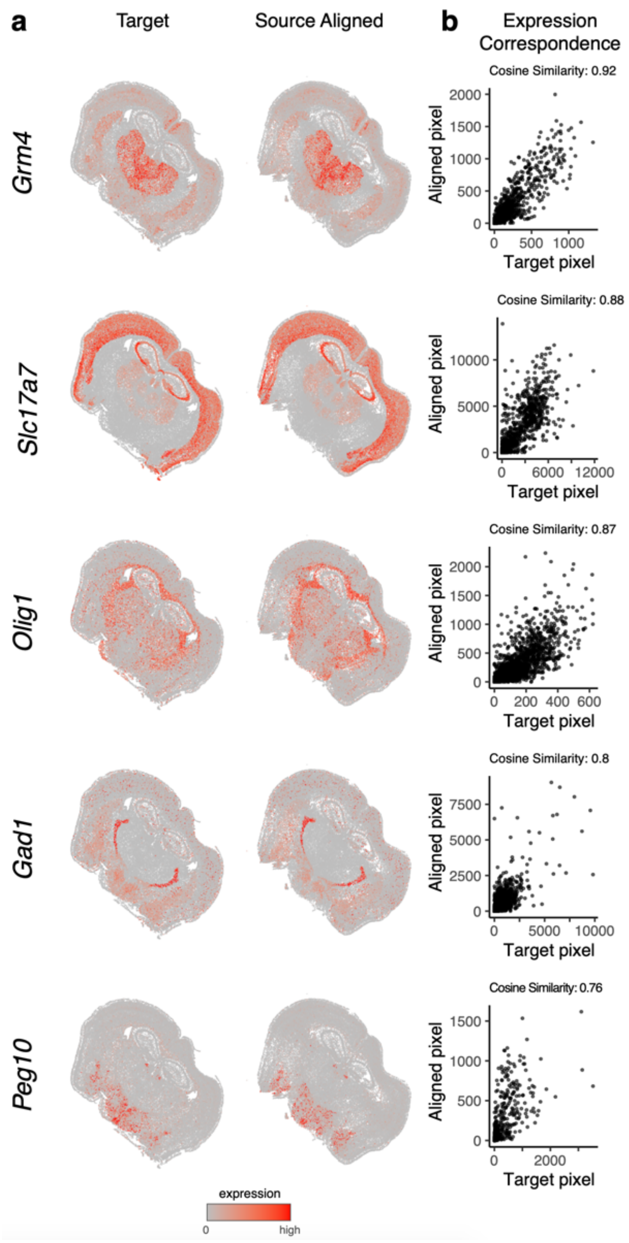

Supplemental Figure 2. ***Additional examples of MERFISH to MERFISH alignment for spatially patterned genes.*** **a.** Correspondence of gene expression spatial organization between the target and aligned source for select spatially patterned genes. **b.** Transcript counts in the target compared to the aligned source at matched pixels for select genes with cosine similarities between transcript counts in target versus aligned source marked.

bioRxiv preprint doi: https://doi.org/10.1101/2023.04.11.534630; this version posted April 12, 2023. The copyright holder for this preprint (which was not certified by peer review) is the author/funder, who has granted bioRxiv a license to display the preprint in perpetuity. It is made available under aCC-BY 4.0 International license.

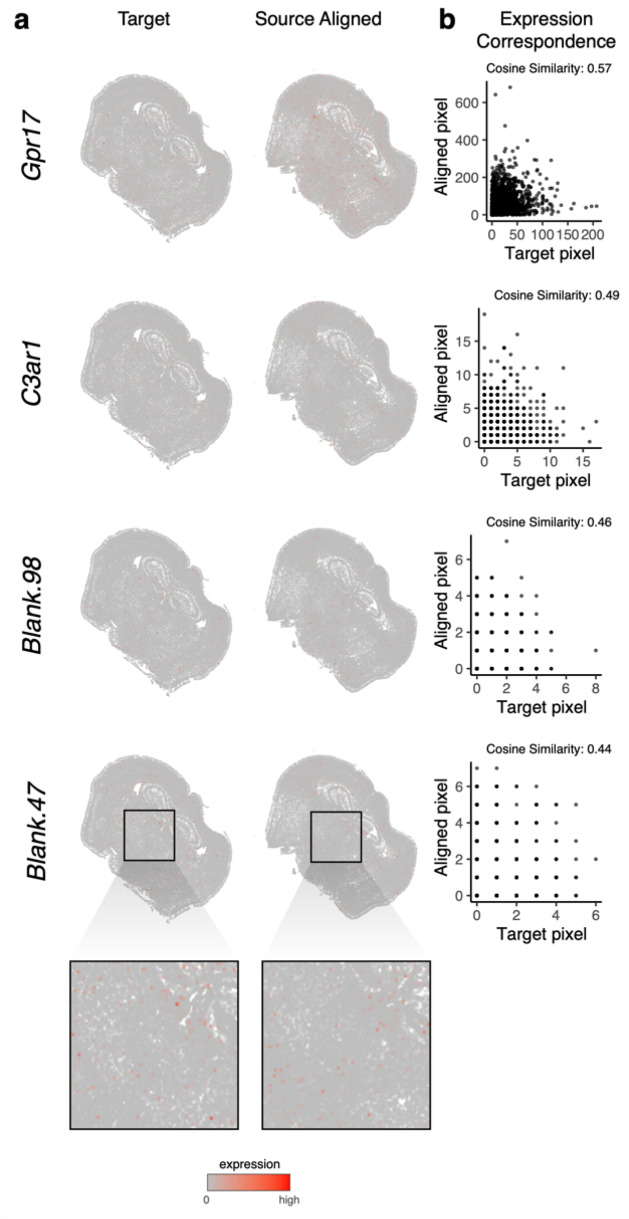

Supplemental Figure 3. ***Additional examples of MERFISH to MERFISH alignment for non-spatially patterned genes.*** **a.** Correspondence of gene expression spatial organization between the target and aligned source for select non-spatially patterned genes (inset displays cells at higher magnification). **b.** Transcript counts in the target compared to the aligned source at matched pixels for select genes with cosine similarities between transcript counts in target versus aligned source marked.

bioRxiv preprint doi: https://doi.org/10.1101/2023.04.11.534630; this version posted April 12, 2023. The copyright holder for this preprint (which was not certified by peer review) is the author/funder, who has granted bioRxiv a license to display the preprint in perpetuity. It is made available under aCC-BY 4.0 International license.

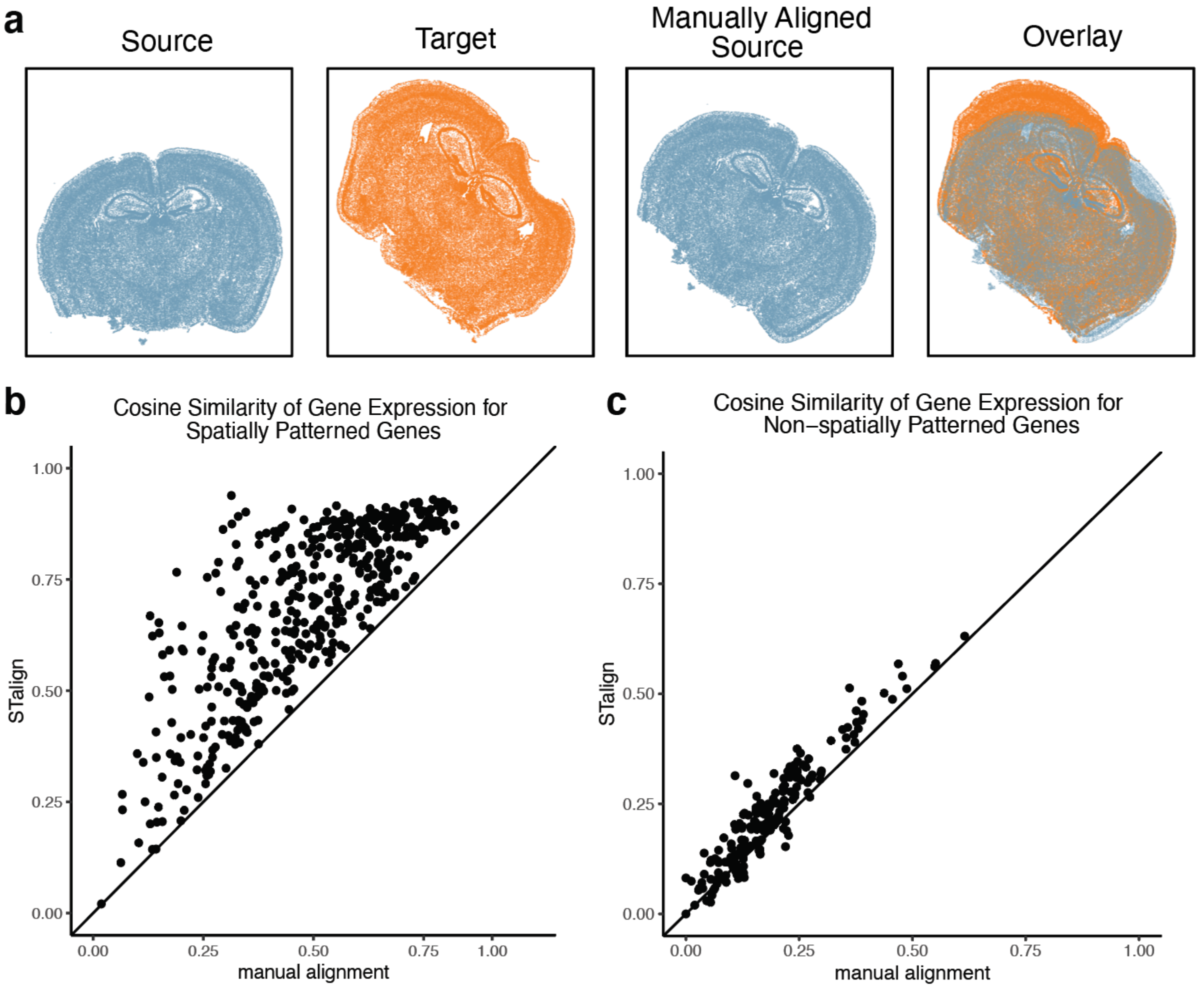

Supplemental Figure 4. ***Evaluation of STalign against manual alignment.*** **a.** Spatial agreement of target and source that has been manually aligned based on simple affine transformations. **b.** Cosine similarities between transcript counts in target versus aligned source for STalign compared to manual alignment for 457 spatially patterned genes. (mean difference = 0.20) **c.** Cosine similarities between transcript counts in target versus aligned source for STalign compared to manual alignment for 192 non-spatially patterned genes. (mean difference = 0.04)

bioRxiv preprint doi: https://doi.org/10.1101/2023.04.11.534630; this version posted April 12, 2023. The copyright holder for this preprint (which was not certified by peer review) is the author/funder, who has granted bioRxiv a license to display the preprint in perpetuity. It is made available under aCC-BY 4.0 International license.

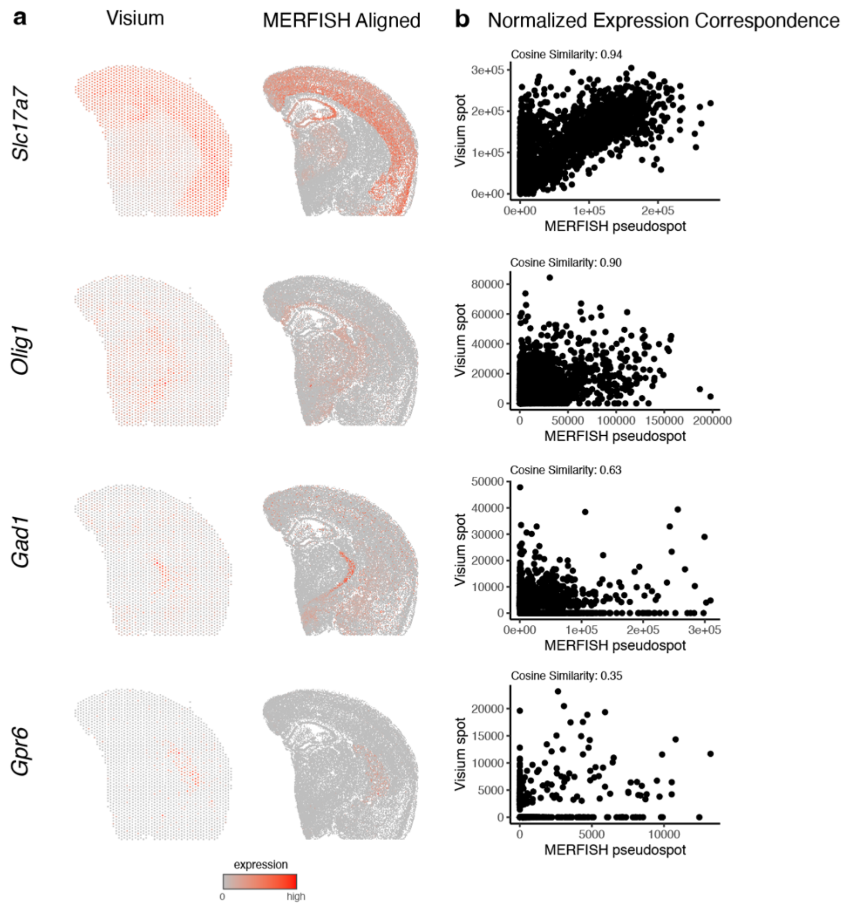

Supplemental Figure 5. ***Additional examples of MERFISH to Visium alignment for spatially patterned genes.*** **a.** Correspondence of gene expression spatial organization between the target and aligned source for select spatially patterned genes. **b.** Normalized gene expression in the Visium target compared to the aligned MERFISH source at matched spots and pseudospots for select genes with cosine similarities between transcript counts in target versus aligned source marked.

bioRxiv preprint doi: https://doi.org/10.1101/2023.04.11.534630; this version posted April 12, 2023. The copyright holder for this preprint (which was not certified by peer review) is the author/funder, who has granted bioRxiv a license to display the preprint in perpetuity. It is made available under aCC-BY 4.0 International license.

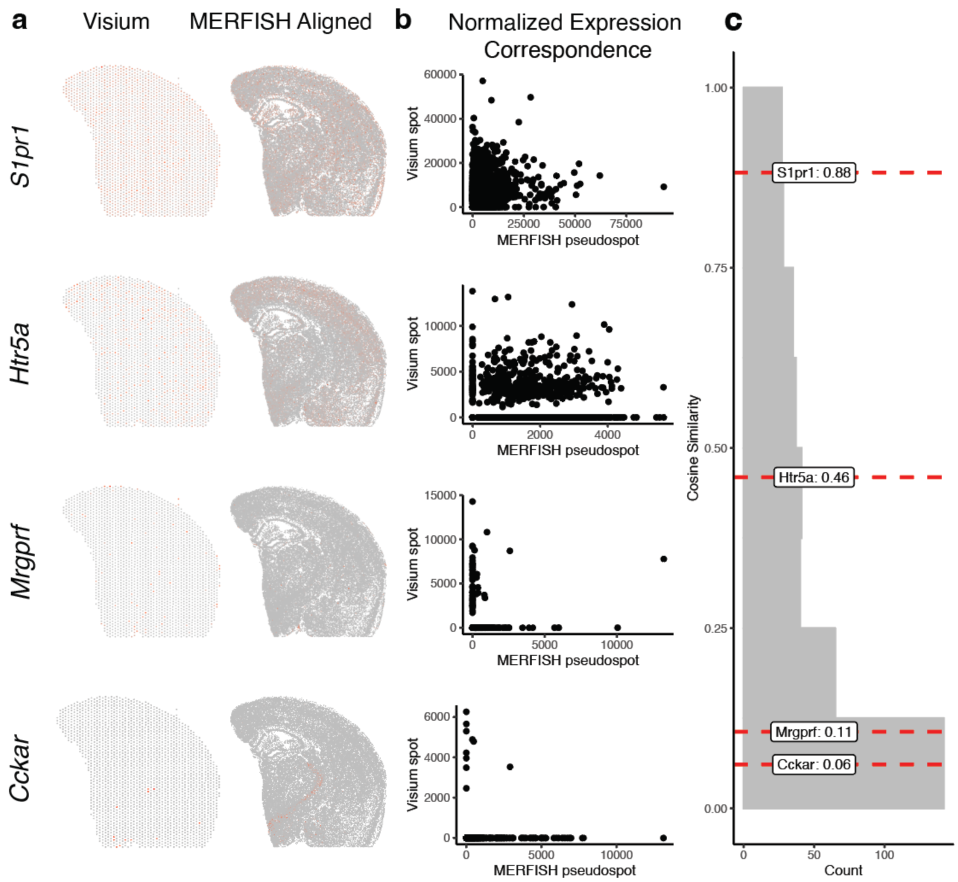

Supplemental Figure 6. ***Examples of MERFISH to Visium alignment for spatially non-patterned genes.*** **a.** Correspondence of gene expression spatial organization between the target and aligned source for select non-spatially patterned genes. **b.** Normalized gene expression in the Visium target compared to the aligned MERFISH source at matched spots and pseudospots for select non spatially patterned genes. **c**. Distribution of cosine similarities between normalized gene expression in the Visium target versus aligned MERFISH source at matched spots and pseudospots for 188 non-spatially patterned genes detected by both ST technologies with select genes marked.

bioRxiv preprint doi: https://doi.org/10.1101/2023.04.11.534630; this version posted April 12, 2023. The copyright holder for this preprint (which was not certified by peer review) is the author/funder, who has granted bioRxiv a license to display the preprint in perpetuity. It is made available under aCC-BY 4.0 International license.

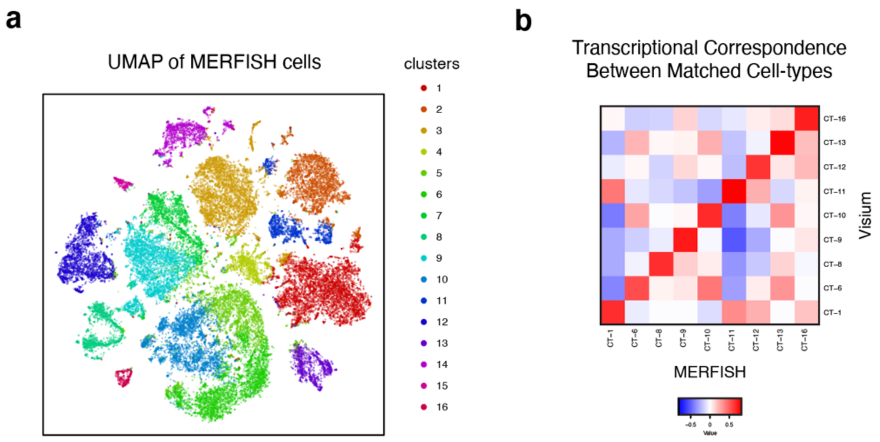

Supplemental Figure 7. ***Cell-type correspondence between clustering of MERFISH data and deconvolution of Visium data*****.** **a.** UMAP embedding of MERFISH cells colored by cluster. **b.** Heatmap of transcriptional correlation between the average expression for MERFISH clusters and deconvolved expression for Visium cell-types from STdeconvolve.

bioRxiv preprint doi: https://doi.org/10.1101/2023.04.11.534630; this version posted April 12, 2023. The copyright holder for this preprint (which was not certified by peer review) is the author/funder, who has granted bioRxiv a license to display the preprint in perpetuity. It is made available under aCC-BY 4.0 International license.

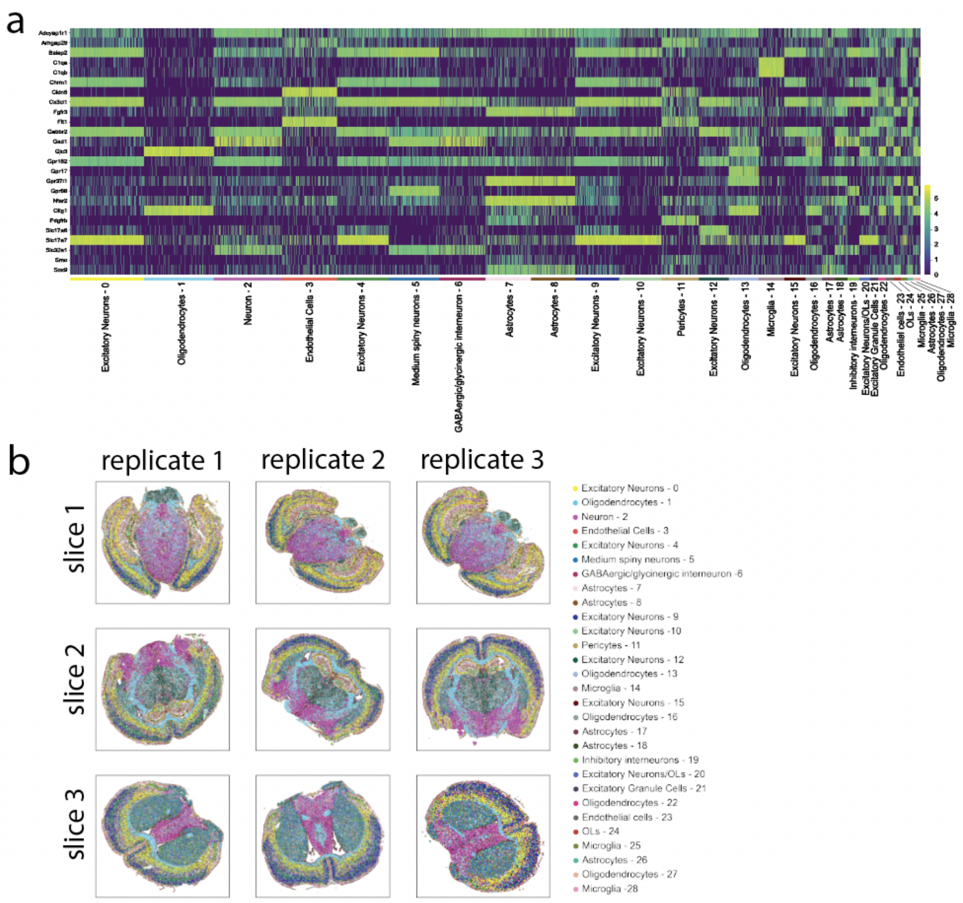

Supplemental Figure 8. C*ell types in MERFISH dataset.* **a.** Heat map of differentially expressed genes in cell types defined by Leiden clustering for all cells across 9 MERFISH datasets. **b.** Cell types defined by differential gene expression and Leiden clustering, represented by different colors, for three biological replicates of three brain slices.

bioRxiv preprint doi: https://doi.org/10.1101/2023.04.11.534630; this version posted April 12, 2023. The copyright holder for this preprint (which was not certified by peer review) is the author/funder, who has granted bioRxiv a license to display the preprint in perpetuity. It is made available under aCC-BY 4.0 International license.

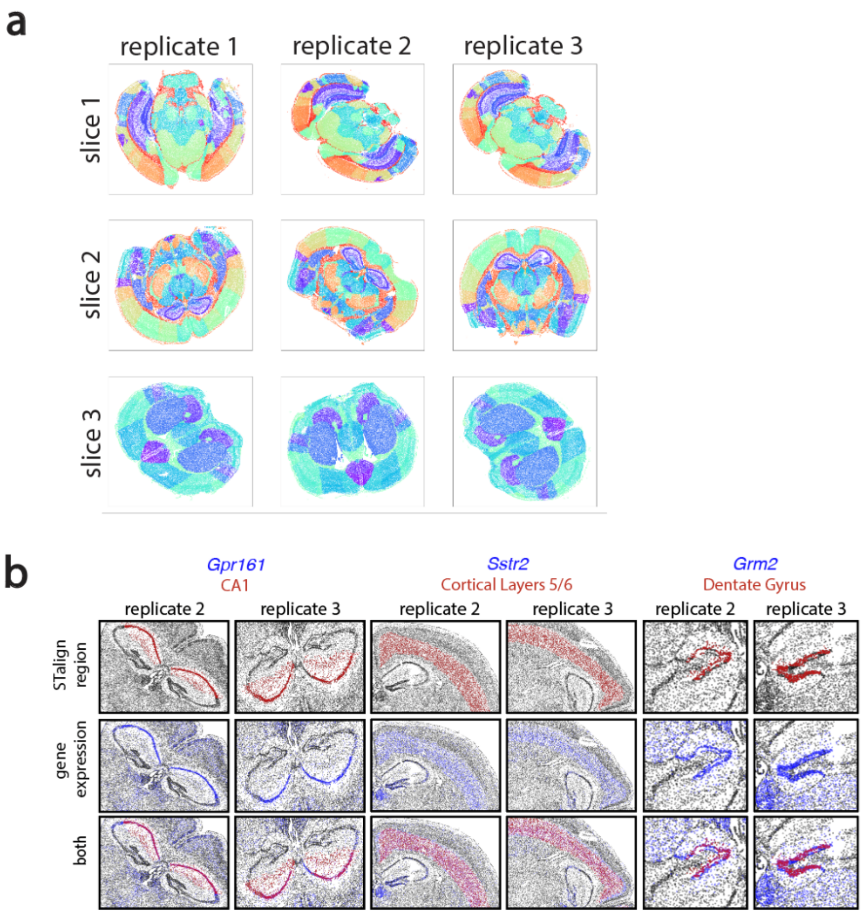

730

731 **Supplemental Figure 9.** ***STalign-defined brain regions.*** **a.** Brain regions defined by STalign,

732 represented by different colors, for three biological replicates of three brain slices. **b.** Examples of

733 genes (blue) expressed in brain regions (red) obtained through 3D alignment of MERFISH slices

734 using STalign. Based on gene expression, brain region annotations show consistency and accuracy

735 across replicates of brain slices.

736

bioRxiv preprint doi: https://doi.org/10.1101/2023.04.11.534630; this version posted April 12, 2023. The copyright holder for this preprint (which was not certified by peer review) is the author/funder, who has granted bioRxiv a license to display the preprint in perpetuity. It is made available under aCC-BY 4.0 International license.

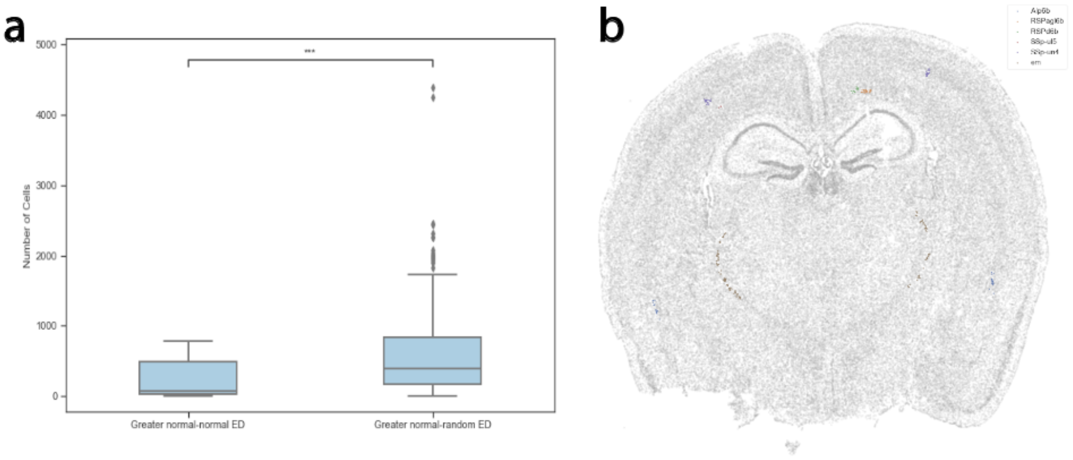

Supplemental Figure 10. ***Analyzing brain regions with lower cell-type compositional similarity between replicates compared to size-matched random regions.*** **a.** Distribution of number of cells in brain regions for which Euclidean-distance (ED) was greater (left) or smaller (right) between replicates compared to matched randomly defined brain regions **b.** Representative MERFISH dataset (Slice 2 Replicate 3) of brain regions in the 'Greater normal-normal ED' suggestive of lower cell-type compositional similarity between replicates compared to size-matched random regions from (a) which were under 50µm in at least one dimension.

bioRxiv preprint doi: https://doi.org/10.1101/2023.04.11.534630; this version posted April 12, 2023. The copyright holder for this preprint (which was not certified by peer review) is the author/funder, who has granted bioRxiv a license to display the preprint in perpetuity. It is made available under aCC-BY 4.0 International license.

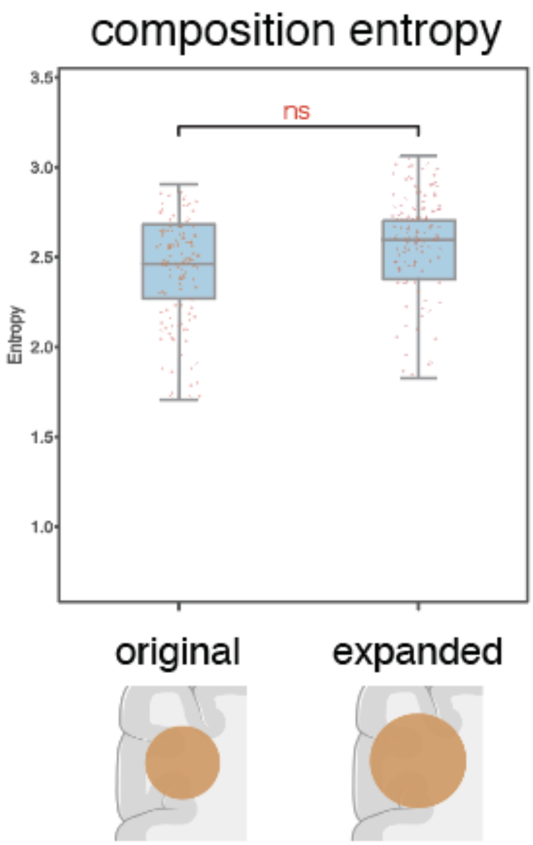

Supplemental Figure 11. ***Entropy for size-matched and expanded random brain regions.*** Non-significant (ns) difference between distribution of cell-type composition entropy for randomly defined brain regions that were matched in size for STalign-defined brain regions (left) versus regions expanded by 100 nearest neighbors (~100μm).

bioRxiv preprint doi: https://doi.org/10.1101/2023.04.11.534630; this version posted April 12, 2023. The copyright holder for this preprint (which was not certified by peer review) is the author/funder, who has granted bioRxiv a license to display the preprint in perpetuity. It is made available under aCC-BY 4.0 International license.

Online Methods for Alignment of spatial transcriptomics data using diffeomorphic metric mapping

Kalen Clifton[1,2,*] Manjari Anant[1,3,*]
Osagie K. Aimiuwu[4] Justus M. Kebschull[2]
Michael I. Miller[2,5] Daniel Tward[6,7] Jean Fan[1,2]

[1]Center for Computational Biology, Whiting School of Engineering, Johns Hopkins University, Baltimore, MD, 21211
[2]Department of Biomedical Engineering, Johns Hopkins University, Baltimore, MD, 21218
[3]Department of Neuroscience, Johns Hopkins University, Baltimore, MD, 21218
[4]University of North Carolina at Chapel Hill, Chapel Hill, NC 27599
[5]Kavli Neuroscience Discovery Institute, The Johns Hopkins University, Baltimore, MD 21211
[6]Department of Computational Medicine, University of California Los Angeles, Los Angeles, CA 90024
[7]Department of Neurology, University of California Los Angeles, Los Angeles, CA 90024

April 11, 2023

1 Methods

1.1 Cell density data model

For single-cell resolution spatially resolved transcriptomics data, we model the point sets of detected cells in the framework of mathematical varifold measures [1]. While the theory extends to more complex spaces of features, here we focus on the locations of cells only, termed the *marginal space measure* (after marginalizing out features other than spatial location) as defined in [2].

Briefly, these space measures are weighted sums of Dirac δ distributions $\rho \doteq \sum_i^{N_q} w_i^\rho \delta_{x_i^\rho}$, where $x_i^\rho \in \mathbb{R}^D$ stores the spatial coordinate of the ith out of N_q cells, and $w_i^\rho \in \mathbb{R}$ stores its weight. In this work, cell positional data is two

bioRxiv preprint doi: https://doi.org/10.1101/2023.04.11.534630; this version posted April 12, 2023. The copyright holder for this preprint (which was not certified by peer review) is the author/funder, who has granted bioRxiv a license to display the preprint in perpetuity. It is made available under aCC-BY 4.0 International license.

dimensional, so $D = 2$, and with some abuse of notation we sometimes write (x, y) instead of x.

We aim to evaluate the similarity between two single-cell resolution spatially resolved transcriptomics datasets, which we call a source and a target. Note other commonly used terms for source are: template, atlas, or moving image, while another commonly used terms for target is: fixed image. To compute distances between datasets, we embed their corresponding space measures ρ_S and ρ_T respectively in the dual of a Reproducing Kernel Hilbert Space V^* and use the standard operator norm (see for example [3]). For some choice of kernel function k, the norm squared is

$$\begin{aligned}\|\rho_S - \rho_T\|^2_{V^*} = &\sum_{i_S,j_S}^{N_{\rho_S}} w^{\rho_S}_{i_S} w^{\rho_S}_{j_S} k(x^{\rho_S}_{i_S}, x^{\rho_S}_{j_S}) \\ &- 2\sum_{i_S}^{N_{\rho_S}} \sum_{j_T}^{N_{\rho_T}} w^{\rho_S}_{i_S} w^{\rho_T}_{j_T} k(x^{\rho_S}_{i_S}, x^{\rho_T}_{j_T}) \\ &+ \sum_{i_T,j_T}^{N_{\rho_T}} w^{\rho_T}_{i_T} w^{\rho_T}_{j_T} k(x^{\rho_T}_{i_T}, x^{\rho_T}_{j_T}) \ . \end{aligned} \quad (1)$$

Here we chose k as a Gaussian with $k(x_i, x_j) = \exp\left(-\frac{1}{2}|x_i - x_j|^2/2\sigma^2\right)$, where $|\cdot|$ denotes the standard Euclidean norm, and σ is a user specified kernel width parameter.

The variables $w^{\rho_S}_{i_S}$, $w^{\rho_S}_{j_S}$, and $x^{\rho_S}_{i_S}, x^{\rho_S}_{j_S}$ corresponds to the weights and spatial coordinates of the ith and jth cells in the source, while $w^{\rho_T}_{i_T}$, $w^{\rho_T}_{j_T}$, $x^{\rho_T}_{i_T}, x^{\rho_T}_{j_T}$ correspond to the weights and spatial coordinates of the cells in the target. For simplicity, in the main paper we write x_S and x_T for source and target points respectively.

In STalign, we initialize weights to 1, though applying nonlinear deformations will modify these weights as discussed below in section 1.4.

1.2 Rasterization

Since computation of this norm is of quadratic complexity in the number of points, we turn to a more efficient representation for computing optimal transformations through rasterization. We can reduce the complexity of our calculations significantly by approximating our space measures through sampling a density signal on a regular grid (known as rasterization), rather than keeping a list of points and weights.

By defining $k^{\frac{1}{2}}$ such that $k^{\frac{1}{2}} * k^{\frac{1}{2}} = k$ (where $*$ refers to convolution), the

bioRxiv preprint doi: https://doi.org/10.1101/2023.04.11.534630; this version posted April 12, 2023. The copyright holder for this preprint (which was not certified by peer review) is the author/funder, who has granted bioRxiv a license to display the preprint in perpetuity. It is made available under aCC-BY 4.0 International license.

above expression for norm squared (1) can be written as

$$\begin{aligned}\|\rho_S - \rho_T\|^2_{V^*} &= \int \left| \sum_{i_S=1}^{N_{\rho_S}} w^{\rho_S}_{i_S} k^{\frac{1}{2}}(x - x^{\rho_S}_{i_S}) - \sum_{i_T=1}^{N_{\rho_T}} w^{\rho_T}_{i_T} k^{\frac{1}{2}}(x - x^{\rho_T}_{i_T}) \right|^2 dx \\ &= \|I^S - I^T\|^2_2 \ . \end{aligned} \tag{2}$$

Note that when k is a radially symmetric Gaussian, $k^{\frac{1}{2}}$ is also a radially symmetric Gaussian but with half the variance. Here we have defined the smooth density function

$$I(x) \doteq [k^{\frac{1}{2}} * \rho](x) = \sum_{i=1}^{N_\rho} w^\rho_i k(x - x^\rho_i) \ , \tag{3}$$

and $\|\cdot\|_2$ is the L_2 norm on functions.

Due to the smoothness introduced by $k^{\frac{1}{2}}$, these functions can be accurately discretized by sampling them on a uniform pixel grid at a resolution rate defined by the user and comparable in size to σ.

Without rasterization, evaluating the function I at a given point would involve a sum over every x_i, an order N complexity operation. After rasterization the function I can be evaluated at any point in order 1 complexity using bilinear interpolation. This allows the norm to be evaluated by summing over a pixel grid in order P complexity (where P is the number of pixels), rather than a double sum over the points x_i in order N^2 complexity.

For example, MERFISH Slice 2 Replicate 3 has 85958 cells, and the rasterized dataset has $336 \times 256 = 86,016$ pixels. The Naive approach would involve 7,388,777,764 terms in the sum (pairs of cells), whereas in the rasterization approach there are only 86,016 terms in the sum (pixels). This is an approximately 86,000 times increase in efficiency which occurs for each iteration of optimization, ignoring the negligible time cost of rasterization itself, which occurs only once at the start of registration.

1.3 Diffeomorphic transformation model

We estimate alignments between two rasterized datasets by applying a transformation $\phi : \mathbb{R}^D \to \mathbb{R}^D$. $\phi(x) \doteq A\varphi_1(x)$, the composition of two transformations: a diffeomorphism (a smooth differentiable transformation with a smooth differentiable inverse) $\varphi_1 : \mathbb{R}^D \to \mathbb{R}^D$ generated in the Large Deformation Metric Mapping (LDDMM) framework [4], and an affine transformation A (i.e. a 3x3 matrix in homogeneous coordinates whose upper left 2x2 block is a linear transform and upper right 2x1 block is a translation vector). In this notation $A\varphi_1(x)$ denotes matrix multiplication of the matrix A and the vector φ_1 in homogeneous coordinates.

In the LDDMM framework a diffeomormphism is generated by integrating a time varying velocity field v_t over time $t \in [0, 1]$, by solving the ordinary

bioRxiv preprint doi: https://doi.org/10.1101/2023.04.11.534630; this version posted April 12, 2023. The copyright holder for this preprint (which was not certified by peer review) is the author/funder, who has granted bioRxiv a license to display the preprint in perpetuity. It is made available under aCC-BY 4.0 International license.

differential equation

$$\frac{d}{dt}\varphi_t = v_t(\varphi_t) \tag{4}$$

with initial condition $\varphi_0(x) = 0$. For identifying alignments, we optimize over v_t rather than φ_1 directly, and to emphasize this dependence we use the superscript φ^v in the main text. Similarly, we use $\phi^{A,v}$ to emphasize the dependence of ϕ on both A and v_t. As long as v_t a smooth function of space, φ_1^v is guaranteed to be diffeomorphic. We enforce this through regularization as described below in section 1.5.

While this section described how we parameterize our transformations, next we need to describe how they act to deform our datasets, in order to use them in an optimization problem.

1.4 Action of transformations on datasets

The action of a transformation ϕ on a space measure dataset ρ moves the spatial coordinate of each cell, and adjusts the weight of each cell based on the transformation's Jacobian determinant.

$$\phi \cdot \rho = \phi \cdot \left(\sum_{i=1}^{N_\rho} w_i^\rho \delta_{x_i^\rho} \right) \tag{5}$$

$$= \sum_{i=1}^{N_\rho} w_i^\rho |d\phi(x_i^\rho)| \delta_{\phi(x_i^\rho)} \tag{6}$$

where $d\phi(x)$ denotes the matrix of partial derivatives of the map ϕ at the point x, and $|\cdot|$ represents the determinant of a matrix.

We note that the standard image action $[\phi \cdot I](x) = I(\varphi^{-1}x)$ has been well studied theoretically (as a left group action), computationally (in terms of its efficient implementation through interpolation), and application-wise (in terms of its use in a variety of image registration platforms e.g. [4]). This image action does not match exactly with the action of a transformation on a space measure dataset (5), although it is a close approximation for datasets of interest. We wish to leverage the existing computational machinery for image actions, and therefore describe the nature of this approximation below:

$$[\phi \cdot I](x) = [\phi \cdot (k^{\frac{1}{2}} * \rho)](x) \tag{7}$$

$$= I(\phi^{-1}(x)) \tag{8}$$

$$= \sum_{i=1}^{N_\rho} w_i^\rho k^{\frac{1}{2}}(\phi^{-1}(x) - x_i^\rho) \tag{9}$$

$$\simeq \sum_{i=1}^{N_\rho} w_i^\rho |d\phi(x_i^\rho)| k^{\frac{1}{2}}(x - \phi(x_i^\rho)) \tag{10}$$

$$= [k^{\frac{1}{2}} * (\phi \cdot \rho)](x) \tag{11}$$

bioRxiv preprint doi: https://doi.org/10.1101/2023.04.11.534630; this version posted April 12, 2023. The copyright holder for this preprint (which was not certified by peer review) is the author/funder, who has granted bioRxiv a license to display the preprint in perpetuity. It is made available under aCC-BY 4.0 International license.

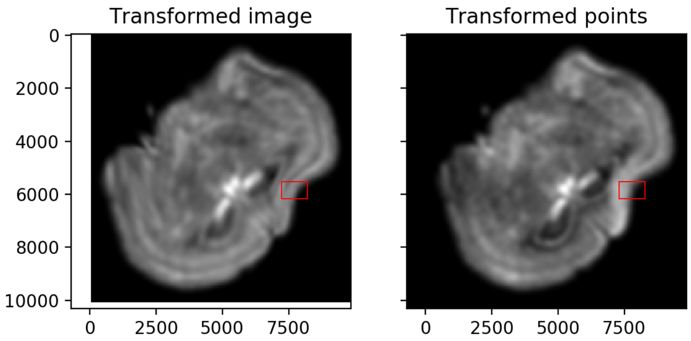

Figure 1: Rasterization followed by deformation with the image action (left), versus naively deforming the positions of cells followed by rasterization (right). Note the intensity changes that occur in regions of high deformation.

The approximate equality is accurate in our examples when $k^{\frac{1}{2}}$ is narrow relative to the smoothness of v_t and wide relative to the spacing between cells. The approximate equality would be exact if $k^{\frac{1}{2}}$ were a Dirac delta function. If cells are too far apart, a larger value of σ could be chosen. For a particularly sparse set of cells, a different method that does not include rasterization would be more appropriate, for example measure matching [5].

With this formulation, deformations can be applied to smooth density images I using interpolation in order P (number of pixels) complexity. Figure 1 illustrates the importance of the Jacobian factor. Without including this factor, transforming a density image alters its brightness, which is typically undesirable: a bigger organ tends to have more cells with the same cell density, rather than the same number of cells with a lower density.

1.5 Image registration

We compute a spatial alignment between two ST datasets by minimizing the sum of two objective functions: a regularization term R, and a matching term M_θ,

$$E(A, v) = R(v) + M_\theta(\phi^{A,v} \cdot I^S, I^T) \tag{12}$$

which we define below. Note that the computation of I is described in section 1.2, the parameterization of $\phi^{A,v}$ is described in 1.3, and the action $\phi^{A,v} \cdot I^S$ is described in the section 1.4. Recall that ϕ depends on both the velocity field v and the affine transform A.

Following the LDDMM framework, we regularize our diffeomorphism via

$$R(v) = \int_0^1 \int_{\mathbb{R}^D} |(\mathrm{id} - a^2\Delta)^p v_t(x)|^2 dx dt \tag{13}$$

bioRxiv preprint doi: https://doi.org/10.1101/2023.04.11.534630; this version posted April 12, 2023. The copyright holder for this preprint (which was not certified by peer review) is the author/funder, who has granted bioRxiv a license to display the preprint in perpetuity. It is made available under aCC-BY 4.0 International license.

where id is an identity matrix, Δ is the Laplacian, a is a constant with units of length that controls spatial smoothness, and $p = 2$ is a power that must be large enough to guarantee that results are diffeomorphisms [6].

Our matching takes the form of [7]

$$M_\theta(\phi^{A,v} \cdot I^S, I^T) = \int_{\mathbb{R}^D} |f_\theta(\phi^{A,v} \cdot I^S](x)) - I^T(x)|^2 W(x) dx \ . \qquad (14)$$

Note that I^T need not correspond to a smooth density image as defined in section 1.2. For example, we include the case where it is a red green blue image corresponding to an H&E stain.

The function f_θ is a transformation of image contrast with unknown parameters θ. We use a polynomial for f_θ, in which case the minimizing parameters θ can be found exactly by solving a weighted least squares problem. The purpose of this transformation is to model differences in contrast between images from the same modality due to calibration issues; and contrast/color differences between different modalities.

The term W is a positive weight that represents the probability that a given pixel in the target image can be matched accurately to one in the source image. For example, if tissue is missing in the target image but not the source image, pixels in the region of missing tissue would get a small weight. Similarly, if the target image included a signal not present in the source (e.g. a bright fluorescence signal).

To optimize E, we alternate between updating W with Gaussian mixture modeling, and jointly updating $(\theta, \phi^{A,v})$ with gradient based methods, using expectation maximization algorithm as discussed in [7]. Recall that updating ϕ corresponds to updating the affine transformation matrix A, and the velocity field v_t which generates the deformation φ_1 from (4).

After solving for the optimal transformation parameters A and v_t, a transformation and its inverse are constructed by solving (4) sampled on a regular grid, using Semi-Lagrangian techniques [8]. With $\phi(x) = A\varphi_1^v(x)$ and $\phi^{-1,A,v}(x) = \varphi_1^{-1,v}(A^{-1}x)$ computed, cell locations $x_{i_S}^{\rho_S}$ in the source image can be mapped into the target by calculating $\phi(x_{i_S}^{\rho_S})$ through linear interpolation. Similarly, a point $x_{i_T}^{\rho_T}$ in the target image can be mapped to the atlas by calculating $\phi^{-1}(x_{i_T}^{\rho_T})$ through linear interpolation.

1.6 3D to 2D alignment

In addition to aligning spatially resolved transcriptomics datasets in which the cell positional information is 2D, we registered the 3D reconstructed Allen common coordinate framework (CCF) atlas (source) to each of the 9 MERFISH datasets (target). The image transformation is similar to the alignment discussed in the section 1.5 with a few exceptions:

It is important to note that all transformations are performed on the source 3D atlas. Since the 50 μm Nissl-stained Allen Brain Atlas CCF v3 was used as the source image, rasterization is not applied to the atlas. The affine transformation A for the 3D-2D alignment is a 4×4 3D matrix in homogeneous

bioRxiv preprint doi: https://doi.org/10.1101/2023.04.11.534630; this version posted April 12, 2023. The copyright holder for this preprint (which was not certified by peer review) is the author/funder, who has granted bioRxiv a license to display the preprint in perpetuity. It is made available under aCC-BY 4.0 International license.

coordinates. The space of dense 3D images in the orbit of the atlas, are defined via diffeomorphisms

$$\phi : (x_1, x_2, x_3) \in \mathbb{R}^3 \to \phi(x) = (\phi_1(x), \phi_2(x), \phi_3(x)) \in \mathbb{R}^3 \tag{15}$$

The diffeomorphism $\phi \in D$ acts on the atlas to generate the orbit of imagery $\mathcal{I}$,

$$I \in \mathcal{I}, I = \phi \cdot I_{atlas} \ . \tag{16}$$

The velocity field v_t is still defined by (4), but $v_t \in \mathbb{R}^3$.

The image I^S in the matching term M represents the transformed source atlas evaluated at $z = 0$, to enable comparison in the same dimension between the source and the target images.

References

[1] W. K. Allard, "On the first variation of a varifold," *Annals of mathematics*, vol. 95, no. 3, pp. 417–491, 1972.

[2] M. Miller, D. Tward, and A. Trouvé, "Hierarchical computational anatomy: unifying the molecular to tissue continuum via measure representations of the brain," *bioRxiv*, pp. 2021–04, 2022.

[3] J. Glaunès, A. Qiu, M. I. Miller, and L. Younes, "Large deformation diffeomorphic metric curve mapping," *International journal of computer vision*, vol. 80, no. 3, pp. 317–336, 2008.

[4] M. F. Beg, M. I. Miller, A. Trouvé, and L. Younes, "Computing large deformation metric mappings via geodesic flows of diffeomorphisms," *International journal of computer vision*, vol. 61, no. 2, pp. 139–157, 2005.

[5] J. Glaunes, A. Trouvé, and L. Younes, "Diffeomorphic matching of distributions: A new approach for unlabelled point-sets and sub-manifolds matching," in *Proceedings of the 2004 IEEE Computer Society Conference on Computer Vision and Pattern Recognition, 2004. CVPR 2004.*, vol. 2, pp. II–II, IEEE, 2004.

[6] P. Dupuis, U. Grenander, and M. I. Miller, "Variational problems on flows of diffeomorphisms for image matching," *Quarterly of applied mathematics*, pp. 587–600, 1998.

[7] D. Tward, T. Brown, Y. Kageyama, J. Patel, Z. Hou, S. Mori, M. Albert, J. Troncoso, and M. Miller, "Diffeomorphic registration with intensity transformation and missing data: Application to 3d digital pathology of alzheimer's disease," *Frontiers in neuroscience*, vol. 14, p. 52, 2020.

[8] A. Staniforth and J. Côté, "Semi-lagrangian integration schemes for atmospheric models—a review," *Monthly weather review*, vol. 119, no. 9, pp. 2206–2223, 1991.
